# The Validity of Quantifying Pulmonary Contusion Extent by Lung Ultrasound Score for Predicting ARDS in Blunt Thoracic Trauma

**DOI:** 10.1155/2022/3124966

**Published:** 2022-05-24

**Authors:** Mohamed Soliman Sayed, Kareem Abdelhamid Elmeslmany, Ahmed Samir Elsawy, Nashwa Abed Mohamed

**Affiliations:** ^1^Critical Care Medicine Department, Cairo University, Cairo, Egypt; ^2^Damanhour Medical Institute Intensive Care Unit, Damanhour, Egypt

## Abstract

**Background:**

Thoracic trauma comprises 20–25% of all traumas worldwide and constitutes the third most common cause of death after abdominal injury and head trauma in polytrauma patients. Pulmonary contusion (PC) is a common injury seen after blunt trauma that is associated with significant morbidity and mortality. The aim of this prospective study was to determine the value of PC extent measurements using lung ultrasound in predicting high risk patients for ARDS development.

**Methods:**

In one year, 50 polytrauma patients with blunt chest trauma were admitted to the ICU at Damanhur Institute. Lung contusion extent was evaluated using a lung US score (LUS) and was compared to the CT contusion score. The ability of the LUS to predict ARDS was tested. The diagnostic accuracy of LUS was compared with chest radiography for lung contusion and pneumothorax with thoracic CT scan as a reference. Patients were restratified by LUS into two groups: severe and nonsevere contusion group. The two groups' data were compared with respect to difference in mortality and injury characteristics.

**Results:**

Lung contusion assessed by LUS score was well correlated to thoracic CT scan measurements (*r* = 0.78). A LUS of 4 was defined as a cut-off value for predicting ARDS development within 72 hours of trauma with sensitivity and specificity (91.67% and 84.21%), respectively. Patients with severe lung contusions had a lower hypoxic index on admission, more ventilator days, a higher risk of ARDS development, more fractured ribs; higher rate of hemothorax and a higher ISS score than patients with nonsevere lung contusions.

**Conclusion:**

LUS on admission can quantify lung contusion extent and the high risk of developing ARDS after blunt thoracic trauma.

## 1. Introduction

Chest trauma is catastrophic event that results in morbidity, incapacity, and mortality in massive number of these trauma victims [1]. The incidence of blunt chest trauma is more than 15% of all trauma admissions to the emergency departments and is the second leading cause of death after head trauma in motor vehicle accidents. Lung contusion is the most frequent thoracic injury in blunt chest trauma, and it is related to elevated morbidity and mortality. Direct damage of the pulmonary tissue causes both local and systemic inflammatory responses that can lead to acute respiratory distress syndrome (ARDS) and more than one organ failure [2]. The initial extent of the lung contusion seems to play a role in these mechanisms. The difficulty in the assessment and management of this patient group arises from the possibility that the patient may additionally develop potentially life-threatening complications up to approximately 72 hrs after injury, even in patients who have sustained what is, first of all, considered a minor injury [3].

This could have significant implications for the clinical prognosis and the selection of appropriate patients for proven therapies [4]. Repeated lung diagnostic evaluations are needed in patients suffering blunt thoracic trauma to follow up on the clinical situation and the results of the therapeutic interventions. Bedside radiography, clinical examination, and serial measurements of respiratory parameters are considered useful, but not sufficient, and computed tomography (CT) is considered the gold standard technique. These patients may need repeated CT chest scanning during their stay in the ICU. Repeated CT scans can be performed without major risks in less severe cases, usually when the patient is not mechanically ventilated; however, transporting a mechanically ventilated patient to the CT suite can be dangerous; several studies have found that even intrahospital transportation of critically ill patients is associated with complications [5]. Also the transport of critically ill trauma patients with associated rib and spine fractures can therefore be a particular challenge and requires time and resources change for the stability of these structures, with possible complications [6].

Lung ultrasound is increasingly being incorporated within the many faces and stages of trauma care and seems to be accurate in this context; bedside ultrasonography seems to be the suitable diagnostic tool. In this prospective study, we aimed to determine the value of PC extent measurements using lung ultrasound score (LUS) in predicting high risk patients for ARDS development after blunt thoracic trauma.

## 2. Methodology

Our study is a prospective cohort study on 50 polytrauma patients admitted to Damanhur Medical Institute ICU with blunt chest trauma during the period from Jan 2018 till Jan 2019, and we excluded pediatric patients (<18 years), penetrating chest trauma, late posttraumatic hospital admission (>24 hrs of trauma), patients with a long lag time between the first lung US and thoracic CT scan (>24 hrs), and patients with surgical emphysema and severe burns.

All selected patients fulfilling the inclusion criteria were subjected to the following on admission: full history, clinical examination, chest X-ray, and routine laboratory investigations. Glasgow coma scale (GCS), revised trauma score (RTS) and injury severity score (ISS), and thorax trauma severity score (TTSS) were calculated. Informed consent was obtained for all patients included in the study. It was obtained either from the patient or his/her healthcare proxy.

**Figure 1:**
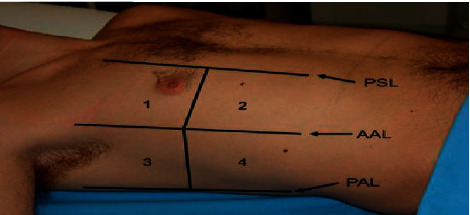
Areas of chest wall examination by ultrasound. PSL: parasternal line; AAL: anterior axillary line; PAL: posterior axillary line (area1–4).

Pulmonary contusion diagnosis and assessment of its extent was evaluated by lung US using a standardized technique imaging on an ultrasound device (Toshiba 35A_590 A) ® curved probe 3.75 MHz. The chest wall was divided into eight areas as seen in Figure 1 with the patient in the supine position. Four quadrants on each side according to the anterior axillary line and the midthoracic line were examined. In each of these areas, sonographic signs of lung contusion were investigated. Lung contusion was diagnosed and calculated by the presence of one of the following criteria: (a) alveolointerstitial syndrome by the presence of multiple B-lines (more than three) originating from pleural line in a person with no clinical cardiopulmonary signs; (b) peripheral parenchymal lesion defined by the presence of C-lines: hypoechoic subpleural focal images with or without pleural line gap.

**Figure 2:**
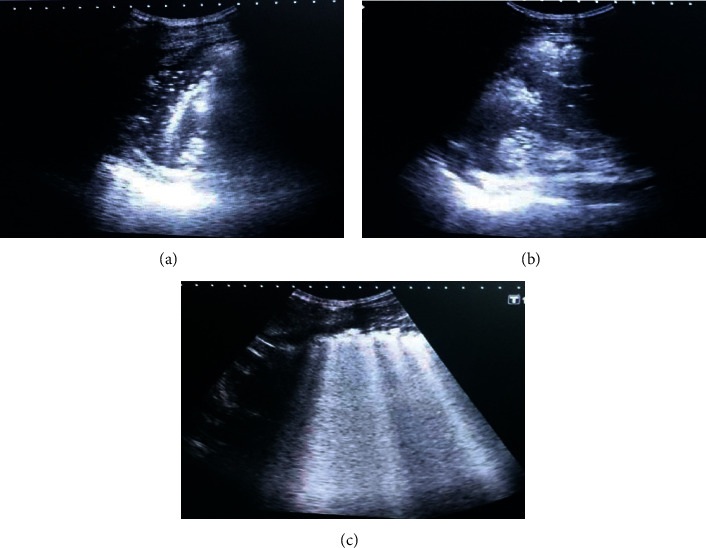
(a) Hypoechoic lesions in examined area filling all the examined area (score 2); (b) hypoechoic lesions in examined area not filling all the examined area (score 1); (c) more than 2 B-lines that were filling all the examined area (score 2).

To assess the extent of lung contusion, we used a LUS defined by Leblanc et al. [7] in each area: 0 = no contusion in the area; 1 = contusion in a part of the area; 2 = contusion in the whole area. By adding the scores of each of the eight areas, we obtained a total score ranging from 0 to 16 per patient (Figure 2).

**Figure 3:**
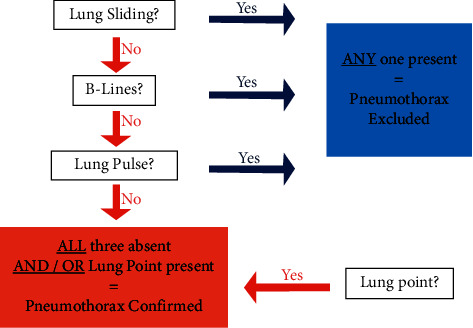
An algorithm for the diagnosis of pneumothorax using LUS.

Diagnosis of pneumothorax was detected using lung ultrasound upon identifying. (a) Absence of lung sliding, which is a horizontal movement of the lung relative to the pleural line. (b) The lack of B lines. (c) Identification of the lung point. We adopted the international recommendations algorithm (Figure 3). We did not assess hemothorax by US because chest tubes were inserted urgently to avoid any delay that could be risky for the patients. If US was done after insertion of the tubes, then results would be conflicting, and technical difficulty was observed.

**Figure 4:**
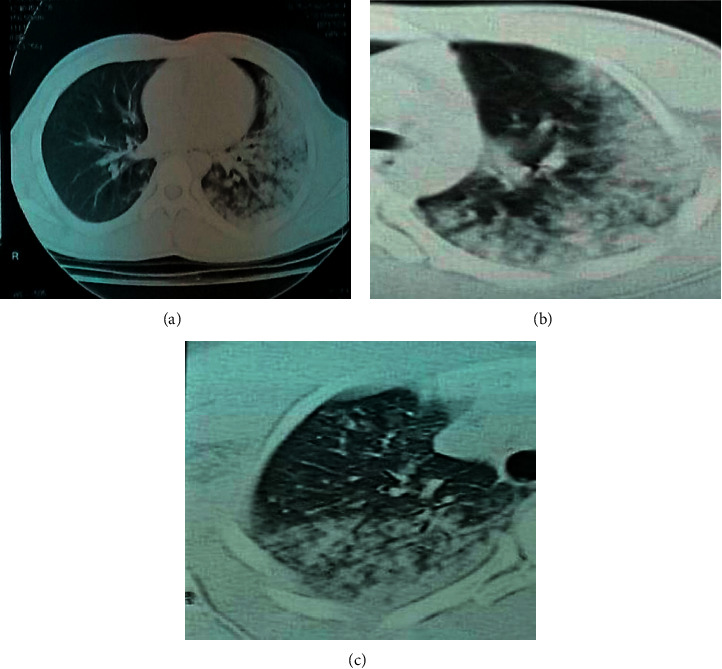
3 CT scan images classifying lung contusions. (a) Total areas on the left lung filled with lesions (score 4); (b) lesions filling the whole lateral and part of medial lung (score 3); (c) contusion not filling all the lateral nor the medial areas (score 2).

Lung contusion on CT scan was described as apical-medial, apical-lateral, medial-basal, and/or lateral-basal in each lung. The following score was applied in each of these areas: 0 = no contusion in the area; 1 = contusion in a part of the area; 2 = contusion in the whole area. We obtained a CT scan score for the extent of lung contusion ranging from 0 to 16 per patient (Figure 4). It is worth nothing notifying that we ignored the occult pneumothorax (anterior, anterolateral, and miniscule) in our study, and so we did not categorise the size of pneumothorax. Thoracic CT scan was performed from the apex of the chest to the diaphragm (using Toshiba scanner, Aquilion prime Model TSX-303a).

The studied patients were observed for the development of ARDS within the first 72 hours after admission. The international Berlin definition [8] for ARDS was used for this purpose. ARDS was staged as mild (PaO2/FiO2 between 201 and 300), moderate (PaO2/FiO2 between 101 and 200), and severe (PaO2/FiO2 less than100). The studied patients were further subdivided in two groups: group A (nonsevere lung contusion) and group B (severe lung contusion) in terms of the identified cut-off point of lung ultrasound score, to correlate the severity of lung contusions and associated injuries, days of mechanical ventilation, trauma scores, and hypoxic index.

All data were fed to the computer and analyzed using IBM SPSS software package version 20.0. (Armonk, NY : IBM Corp.). Qualitative data were described using number and percent. The Kolmogorov-Smirnov test was used to verify the normality of distribution. Quantitative data were described using range (minimum and maximum), mean, standard deviation, median, and interquartile range (IQR). The significance of the obtained results was judged at the 5% level.

## 3. Results

Our study enrolled 50 patients admitted by polytrauma with blunt chest trauma in the ICU unit; the mean age was 35.74 ± 15.17 years. At the time of the study, 82% of the population was male, compared with 18% female. The most common mode of trauma (70%) was a car accident, followed by a fall from height (20%) and a direct impact (10%). Extremity bone fractures were the most common associated injury (58%), followed by head trauma (42%), abdominal trauma (32%), pelvic fractures (12%), and spinal fractures (12%) (Table 1).

The systolic arterial blood pressure ranged from 40 to 150 mm·Hg, with a mean of 82.20 ± 40.37 mm·Hg. The hypoxic index (P/F ratio) at admission ranged from 55.0 to 460.0, with a mean of 213.26 ± 85.57. The Glasgow Coma Scale (GCS) ranged from 3.0 to 15.0 with a mean of 11.54 ± 3.67, Revised Trauma Score (RTS) ranged from 2.0 to 8.0, with a mean of 6.44 ± 1.61, Injury Severity Score (ISS) ranged from 16.0 to 58.0, with a mean of 33.42 ± 11.51, and Thorax Trauma severity score (TTSS) ranged from 2.0 to 14.0, with a mean of 7.84 ± 3.20 (Table 1).

Pneumothorax in our study was detected in 24 cases (48%), hemothorax was seen in 21 cases (42%), rib fractures were detected in 26 cases (52%), two to three fracture ribs were seen in five cases (10%), more than three fracture ribs were seen in 16 cases (32%), and flail chest was seen in five cases (10%). The need of invasive mechanical ventilation was found in 42 cases (84%). The range of days of mechanical ventilation was ranged from 3.0–40.0 days with mean of 12.05 ± 8.64 days and median of 10 days. Regarding the outcome, 12 cases (24%) developed ARDS, while the other 38 cases (76%) did not develop ARDS. Mild ARDS was found in one case (2%), moderate ARDS was found in seven cases (14%), and severe ARDS was found in four cases (8%). Regarding mortality within 28 days, in our study, 37 cases survived (74%) and 13 patients died (26%).

**Figure 5:**
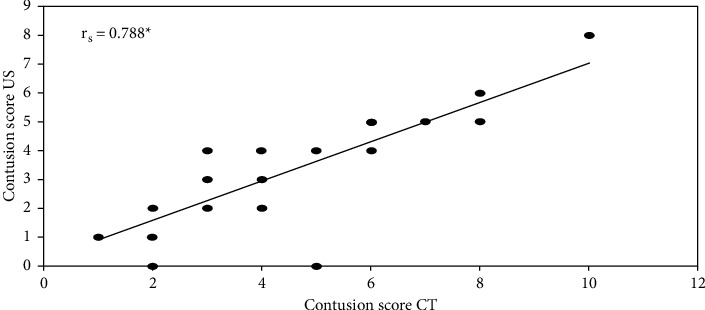
Correlation between CT and US according to lung contusion volume score. R = 0.781.

Regarding lung ultrasound comparison to CT scan, the sensitivity in detecting pneumothorax in our study was 83.33%, specificity was 100.0%, PPV was 100.0%, NPV was 86.67%, and accuracy was 92.0% (Table 2). Diagnosis of lung contusion using LUS was obtained in 94% of the studied cases, while chest X-ray diagnosis was obtained in 52% of the studied cases compared with the CT chest. By comparing CT score and LUS score in terms of the lung contusion volume, CT lung contusion score ranged from 1.0 to 10.0, with a mean 3.94 ± 2.03 while the LUS contusion score was ranged from 0.0 to 8.0, with a mean 2.92 ± 1.68. There was a good positive correlation between the two scores, with a Spearman coefficient of 0.781 (Figure 5).

**Figure 6:**
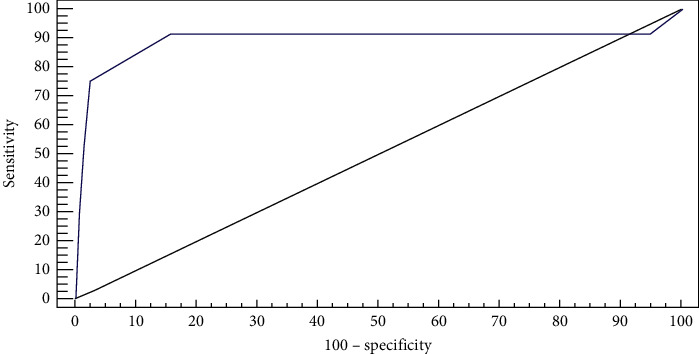
ROC curve for lung contusion score to predict ARDS.

**Figure 7:**
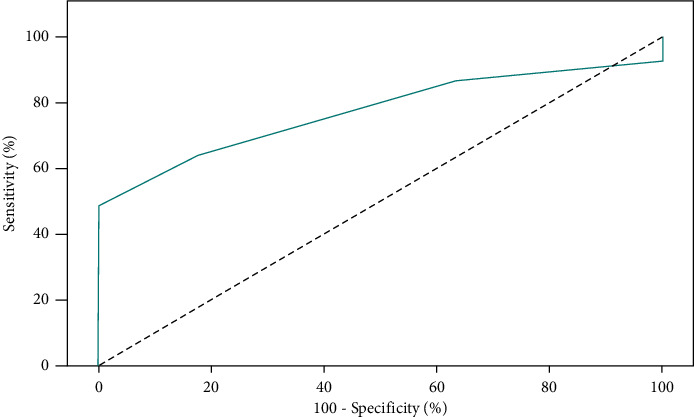
ROC curve for contusion score US to discriminate ISS (>24).

Our results shows that LUS score of four or more of 16 was identified as a cut-off value for predicting ARDS with sensitivity of 91.67%, specificity of 84.21%, PPV of 64.7%, and NPV of 97.0% (Table 3; Figure 6). Multiple cut-off values were measured for each outcome (hypoxic index (<150), mechanical ventilation days (>7 days), trauma scores and associated injuries); for the HI (<150) no cut-off value could be calculated (*P*=0.854); for MV > 7 days no cut-off value could be calculated (*P*=0.15); for ISS>24 cut-off value (>2) was identified (*P*=0.006); associated injuries >2 organs no cut-off value can be calculated (*P*=0.85) (Figure 7).

Patients were further stratified by lung ultrasound contusion score into a severe group (LUS ≥ 4) or nonsevere group (LUS˂4). As reported in Table 4, the two groups did not differ significantly in age, gender, GCS, and mortality rates (*P* > 0.05). A trend was evident in the severe contusion group toward a higher incidence of hemothorax (*p* < 0.001), lower hypoxic index (*P*=0.010), higher incidence of ARDS (*p* < 0.001), and more than three fracture ribs (*P*=0.014), and more ventilator days (*p* < 0.001) and higher ISS (*P*=0.010) were detected in the severe group, with a significant statistical difference (Table 5).

When the severe and nonsevere lung contusion groups were compared in terms of ARDS development, only one patient (3.2%) in the nonsevere group was complicated by moderate ARDS, whereas 11 patients (31.6%) in the severe group were complicated by ARDS (one patient with mild ARDS; seven patients with moderate ARDS; four patients with severe ARDS) with a significant *P* value (0.001) (Table 5).

By comparing the nonsevere and severe lung contusion groups regarding the need for invasive mechanical ventilation, 27 (87.1%) patients in nonsevere group were indicated for invasive ventilation, while in the severe group, 15 patients (78.1%) were indicated for mechanical ventilation without significant difference between the two groups (p = 0.459). The range of days of mechanical ventilation in nonsevere group was 3–15 days with a mean of 8.0 ± 3.11 days, while in severe group, the range was 4–37 days, with a mean of 18.47 ± 9.02 days with a significant difference between the two groups (*P* value > 0.001) (Table 4).

By comparing the two groups regarding mortality, eight patients (25.8%) died in nonsevere lung contusion score group, while five patients (26.3%) died in severe lung contusion score group, with no significant difference between the two groups (Table 6).

## 4. Discussion

Pulmonary contusion is a significant problem in patients with blunt trauma, being the most detected intrathoracic injury in this patient population. Accurate quantification of lung contusion extent is central to its understanding. Plain chest radiography is known to underestimate the actual degree of pulmonary injury [9]; computed tomography (CT) is considered the gold standard technique, but it is associated with a significant increase in total costs and exposure to radiation. Furthermore, severe trauma causes circulatory failure in the initial phase that contraindicates the transfer to CT scan. In this regard, ultrasound has been considered as an alternative diagnostic tool. It is known that the extension of the lung contusion, compared with the total lung volume, clearly correlates to the risk of ARDS. These data reinforce the need for a more sensitive method to diagnose early lung contusions, allowing the critical care physician to more accurately preview the clinical course and, eventually, modify intervention (e.g., fluid restriction, prehospital triage, and early admission to the ICU) [10].

Contusion is characterized by parenchymal injuries and accumulation of blood and fluid in the lung tissues. These tissues lie in the deep layers of the chest cavity, and so, the penetrating power of ultrasound wave is more helpful than the image resolution (which is directly related to the wave's frequency). Application of transducers with frequencies less than 5 MHz yields better diagnostic values compared with higher [11]. This is also related to the nature of the lesion. In our study, we used a transducer with a frequency of 3.75 MHz. We excluded patients with subcutaneous emphysema as subcutaneous gas interferes with the sonographic waves, and this may prevent the accurate diagnosis of underlying lesions and can underestimate the lesions and the scores calculated.

Our results showed that lung ultrasound was well correlated with CT in the assessment of lung contusion extent and was superior to chest radiography in contusion diagnosis. We used the same lung ultrasound score that was suggested by Leblanc et al. [7] and found that the extent of lung contusion assessed by LUS score was well correlated with thoracic CT scan measurements with Spearman coefficient of 0.78. Leblanc et al. [7] reported that the extent of lung contusion was correlated with thoracic CT scan measurements with a Spearman's coefficient of 0.82. In a small cohort, Rocco et al. [6] addressed this issue on 12 trauma patients to evaluate the role of LUS compared with CT scan and bedside CXR in the evaluation of trauma patients; they reported that the number of lung contusion areas measured using CT well correlated with the extent of lung injury measured by lung US (correlation coefficient = 0.86).

In our study, ARDS was diagnosed in 12 cases (24%). Mild ARDS was found in one case (2%), moderate ARDS was found in seven cases (14%), and severe ARDS was found in four cases (8%). We defined a lung ultrasound score of four or more of 16 (about 25% of the total score) as a cut-off value for predicting ARDS development within 72 hrs of trauma with sensitivity 91.67%, specificity 84.21%, PPV 64.7%, and NPV 97.0%. Leblanc et al. [7] reported that a LUS score of six or more of 16 was predictive of ARDS. The difference between our results and Leblanc et al.'s results [7] may be related to the increased rate of pneumothorax in our study. Additionally, lung US is also an operator dependent procedure, which could explain this small difference. In our study, the time between trauma and the development of ARDS ranged from 20.0 to 65.0 hours, with a mean of 39.25 ± 12.88 hours and median of 40.0 hours (31.0–44.0). Haider et al. [12] reported that the mean time to develop ARDS was two days after trauma onset and ranged from one to four days in a study of 288 patients to determine the frequency of thoracic trauma and ARDS in polytrauma patients and evaluate the impact of thoracic trauma on the occurrence and the onset of ARDS. In this study, we assessed the diagnostic performance of lung US in pneumothorax compared with CT chest. The sensitivity of lung US in detecting pneumothorax was 83.33%, specificity was 100.0%, positive predictive value (PPV) was 100.0%, negative predictive value (NPV) was 86.67%, and accuracy was 92.0%. This was in concordance with the meta-analytic study of Ebrahimi et al. [13], which showed ultrasound accuracy in detection of pneumothorax, with a sensitivity of 87% and specificity of 99%. We further stratified the patients by lung ultrasound contusion score into a nonsevere group (LUS˂4) and severe group (LUS≥4), according to the calculated cut-off value of our study. We found that lung ultrasound may allow identification of high-risk patients for ARDS and mortality. Additionally, patients with severe contusion had a higher incidence of hemothorax, lower Pao2/Fio2 ratio, and higher incidence for ARDS. It was also associated with more than three fracture ribs, more ventilator days, and higher ISS with a significant difference between the two groups.

Strumwasser et al. [14] reported that lung contusion greater than 20% of total lung volume specifically identified patients at risk for developing complications. Hamrick et al. [15] found that duration of mechanical ventilation correlated with the contusion volume >20% of total lung volume and could be used to identify high-risk patients. Consistent with our results, Mahmood et al. [4] suggested that, in addition to prolonged mechanical ventilation, patients who have severe lung contusion are at increased risk of developing ARDS and a lower PaO2/FiO2 ratio. Tyburski et al. [16] quantified the PC and observed that severe PC was correlated with lower PaO2 : FiO2 ratio and higher ISS. On the other side, Mahmood et al. [4] reported that contusion volume did not predict total ventilator days suggesting that outcomes for patients with polytrauma and lung contusion are multifactorial in nature. Wang et al. [17] reported that admission Pao2/Fio2 ratio and the PC volume are not linearly correlated, and the admission Pao2/Fio2 ratio may be affected by other factors, such as the state of consciousness.

In our study, there was no significant difference in mortality between the severe lung contusion and nonsevere contusion group denoting that lung contusion is not the only factor associated with mortality, and it is mostly a multifactorial process. We then observed the patients for 28 days, 37 cases were survivors (74%), and 13 patients were nonsurvivors (26%). This outcome is concordant with that of Pehlivanlar et al. [18] who reported that mortality occurred in 159 (28.1%) cases, while 405 (71.8%) were discharged from the ICU. Disagreement to our results Huber et al. [19] who conducted a study on 22613 trauma patients aiming to identify the influence of critical structural damages in patients with blunt chest trauma on mortality; they reported that 82.5% of cases were survivors and 17.5% of cases were nonsurvivors. Wang et al. [17] reported that there was no significant difference between the two groups (severe and nonsevere lung contusion) regarding mortality. On the other side, Deunk et al. [20] reported that there was a significant difference between the two groups (severe and nonsevere lung contusion) regarding mortality. Head injury and abdominal trauma are the most common cause of mortality in polytrauma patients, and this may be the possible explanation of this big controversy, but this hypothesis needs further powerful studies for proper evaluation with much higher numbers of included patients.

In this study, the range of ISS score in nonsurvivors patients was 16.0–59.0, with a mean of 41.62 ± 10.86, while in the survivors patients, the range was 16.0–59.0, with a mean of 30.54 ± 10.40, with a significant difference between the two groups (*P* value = 0.002). RTS score in this study was not related to mortality. Similar to our results, Akhavan and Mohammadian [21], who conducted a study on 70 polytrauma patients aiming to evaluate the performance of ISS and RTS scoring systems in trauma patients, reported that ISS scoring system performed better than the RTS in predicting of mortality. In contrast to these results, Rizk et al. [22], who conducted a study on fifty patients aiming to find a relation between initial scores and their outcome, reported that RTS was better than ISS in predicting mortality among polytrauma patients. This may be due to that the patients with similar injury severity score may have totally different RTS score.

## 5. Conclusion

Ultrasound is of great value and is correlated with the gold standard CT for diagnosis and quantification of lung contusion extent. Lung US is a sensitive test to diagnose pneumothorax in relation to thoracic CT. In the severe lung contusion group, our study revealed a less hypoxic index on admission, more ventilator days, higher risk of ARDS development, more rib fractures, higher incidence of hemothorax, and a higher ISS score than nonsevere lung contusions. Lung ultrasound can detect high risk patients for development of ARDS within 72 hours in blunt chest trauma. According to our results, a LUS score of four or more was the cut-off value to determine the high-risk patients (sensitivity 91.67% and specificity 84.21%).

## Figures and Tables

**Table 1:** Patient characteristics and scores.

Patient characteristic	Value (*n* = 50)
Age, y	35.74 ± 15.17
Male, n (%)	41 (82%)
Physiologic parameters	
Lactate	2.99 ± 1.38
SBP	82.20 ± 40.37
Pao2/Fio2	213.26 ± 85.57
Mode of trauma	
Rta, n (%)	35 (70%)
Falling from height, n (%)	10 (20%)
Direct impact, n (%)	15 (30%)
Distribution of injuries	
Head trauma, n (%)	21 (42%)
Abdominal trauma, n (%)	16 (32%)
Spinal fractures, n (%)	6 (12%)
Extremity bone fractures, n (%)	29 (58%)
Pelvic fracture, n (%)	6 (12%)
Injury severity measures	
GCS	11.54 ± 3.67
ISS	33.42 ± 11.51
RTS	6.44 ± 1.61
TTSS	7.84 ± 3.20
Associated thoracic injuries	
Pneumothorax, n (%)	24 (48%)
Hemothorax, n (%)	21 (42%)
Fractured ribs, n (%)	26 (52%)
Flail chest, n (%)	5 (10%)
Pulmonary status and outcome	
ARDS, n (%)	12 (24%)
Ventilator days	12.05 ± 8.64
Mortality, n (%)	13 (26%)

**Table 2:** Sensitivity, specificity, and accuracy for US pneumothorax diagnosis to CT.

Pneumothorax US	Pneumothorax				Sensitivity	Specificity	PPV	NPV	Accuracy
	No (*n* = 26)		Yes (*n* = 24)						
	No.	%	No	%					
No	26	100.0	4	16.7	83.33	100.0	100.0	86.67	92.0
Yes	0	0.0	20	83.3					

**Table 3:** Sensitivity and specificity tests for lung contusion score to predict the development of ARDS.

Contusion score	AUC	*P*	95% C. I		Cut off	Sensitivity	Specificity	PPV	NPV
			LL	UL					
US	0.896	<0.001	0.743	1.00	≥4	91.67	84.21	64.7	97.0

**Table 4:** Severe and nonsevere lung contusion group characteristics.

Variables	Group A Nonsevere LUS score <4 (*n* = 31)	Group B Severe LUS score ≥4 (*n* = 19)	*P*-value
Age, yr	36.55 ± 15.36	34.42 ± 15.18	0.652
Male, n	24	17	0.452
Female, n	7	2	
Pao_2_/Fio_2_	236.45 ± 87.17	176.53 ± 68.63	0.010^*∗*^
GCS	11.35 ± 4.07	11.84 ± 2.99	0.943
ISS	30.19 ± 10.69	36.68 ± 11.09	0.010^*∗*^
Head trauma, n	14	7	0.563
Abdominal trauma, n	9	7	0.566
Extremity bone fractures, n	19	10	0.547
Pelvic fracture, n	4	2	1.000
Spinal fractures, n	2	4	0.184
Hemothorax, n	7	14	<0.001^*∗*^
2-3Fractured ribs, n	7	3	0.722
˃3 fractured ribs, n	6	10	<0.014^*∗*^
ARDS incidence, n	1	11	<0.001^*∗*^
Ventilator days	8.0 ± 3.11	18.47 ± 9.02	<0.001^*∗*^
Mortality, n	8	5	1.000

**Table 5:** Relation between LUS contusion score and development of ARDS.

Patients developed ARDS	Contusion score US				*χ* ^2^	MC_P_
	Group A score<4 (*n* = 31)		Group B score≥4 (*n* = 19)			
	No.	%	No.	%		
No	30	96.8	8	42.1	18.554^*∗*^	<0.001^*∗*^
Mild	0	0.0	1	5.3		
Moderate	1	3.2	6	31.6		
Severe	0	0.0	4	21.1		

**Table 6:** The severity of lung contusion and mortality.

	Contusion score US				Test of sig.	*P*
	Group A score <4 (*n* = 31)		Group B score ≥4 (*n* = 19)			
	No.	%	No.	%		
Outcome						
Nonsurvivors	8	0.0	1	5.3	X^2^ = 0.002	FE_P_ = 1.000
survivors	23	74.2	14	73.7		

## Data Availability

The data used to support the findings including patients and procedures of this study are available from the corresponding author upon request.
